# In Vitro Bioaccessibility and Functional Properties of Phenolic Compounds from Enriched Beverages Based on Cocoa Bean Shell

**DOI:** 10.3390/foods9060715

**Published:** 2020-06-02

**Authors:** Carolina Cantele, Olga Rojo-Poveda, Marta Bertolino, Daniela Ghirardello, Vladimiro Cardenia, Letricia Barbosa-Pereira, Giuseppe Zeppa

**Affiliations:** 1 Department of Agricultural, Forest and Food Sciences (DISAFA), University of Turin, 10095 Grugliasco, Italy; carolina.cantele@unito.it (C.C.); olgapaloma.rojopoveda@unito.it (O.R.-P.); daniela.ghirardello@unito.it (D.G.); vladimiro.cardenia@unito.it (V.C.); letricia.barbosapereira@unito.it (L.B.-P.); giuseppe.zeppa@unito.it (G.Z.); 2 RD3 Department-Unit of Pharmacognosy, Bioanalysis and Drug Discovery, Faculty of Pharmacy, Université libre de Bruxelles, 1050 Brussels, Belgium; 3 Department of Analytical Chemistry, Nutrition and Food Science, Faculty of Pharmacy, University of Santiago de Compostela, 15782 Santiago de Compostela, Spain

**Keywords:** cocoa bean shell, by-products, polyphenols, bioaccessibility, capsule, tea bag, antiradical activity, α-glucosidase inhibition

## Abstract

The cocoa bean shell (CBS), a cocoa by-product, contains a significant number of bioactive compounds with functional properties, such as polyphenols and methylxanthines, and is used as an ingredient in beverages and foods. In this work, the bioaccessibility of polyphenols and methylxanthines after In Vitro digestion was evaluated in new flavoured beverages for at-home consumption (capsules and tea bags). In addition, the polyphenolic composition, functional properties (antiradical and α-glucosidase inhibition capacities) and consumer acceptability of these beverages were evaluated. In both capsule and tea bag beverages, the bioaccessibility of methylxanthines was 100% while that of total polyphenols exceeded 50%. The main polyphenols determined using reverse-phase liquid chromatography were type B procyanidins and epicatechin. The antiradical activity in capsule and tea bag beverages was 1.75 and 1.88 mM of Trolox equivalents, respectively, of which 59.50% and 57.09% were recovered after simulated digestion. The percentage of α-glucosidase inhibition before In Vitro digestion (51.64% and 53.82% for capsules and tea bags, respectively) was comparable to that of acarbose at 0.5 mM. All the beverages obtained a high consumer acceptability. Therefore, these results highlight that CBSs can be used as a valid source of bioactive compounds in the preparation of beverages with homemade techniques.

## 1. Introduction

The chocolate industry generates large amounts of by-products, as the cocoa bean (CB) represents one-third of the total weight of the fruit while the remaining 67% is made up of the pod husk, placenta, shell, and germ [1]. The cocoa bean shell (CBS) is the thin and fibrous external tegument of the bean, which is removed with the germ before or after roasting [2]. The CBS represents 10–17% of the bean and, considering 4.7 million tons of cocoa beans are processed worldwide each year, its annual production amount is approximately 700,000 tons that are generally intended to be used as fuel, feed, or fertilizer [3]. The CBS has an interesting nutritional profile with more than 50% (*w*/*w*) of dietary fibre [2], almost three times higher than the CB. It also has a low fat content with physical and chemical characteristics identical to those of cocoa butter except for linoleic acid, which is higher in the CBS (7.49% in CBS fat vs. 3.93% in cocoa butter) [2,4]. Moreover, the CBS is also a source of dietary minerals (e.g., calcium, iron, and magnesium) [5] and key aromatic compounds (e.g., 3-methylbutanal and 2,3,5-trimethylpyrazine) that contribute to its cocoa flavour notes [6,7]. Finally, the CBS contains a high number of polyphenols with a total phenolic content that ranges from 3.12–94.95 mg of gallic acid equivalents (GAE)/g of dried CBSs mainly resulting from flavanols, which include catechins, epicatechins, and procyanidins [3]. Recently, polyphenols have been widely studied for their health benefits, serving as antioxidants, chelators of bivalent cations, and enzyme activity inhibitors (e.g., enzymes responsible for reactive oxygen species (ROS) production) and modulators (e.g., nitric oxide synthase, cyclooxygenase, and lipoxygenase) [8]. Skenderidis et al. [9] demonstrated the capability of polyphenols from goji berry extract to enhance the antioxidant status of muscle cells C2C12 by increasing the levels of a crucial antioxidant molecule, the glutathione, and the protective effects against radical-induced DNA damage.

Many biofunctionalities and potential health benefits of cocoa polyphenols have been reviewed by Rojo-Poveda et al. [3], such as antidiabetic activity; anticariogenic, anticarcinogenic, and anti-inflammatory effects; and actions on cardiovascular health. In particular, flavonoids have been suggested as natural inhibitors of the α-amylase and α-glucosidase enzymes directly involved in the degradation of complex carbohydrates; in other words, they are able to lower the amount of glucose intake directly binding to amino acid residues in the enzyme active sites and exclude substrate binding [10]. The inhibition capacity of flavonoids depends on their molecular structure and is higher when planar structure of the rings, double bonds in the 2 and 3 positions in the C_3_ part of the flavonoid skeleton (C_6_–C_3_–C_6_), and hydroxylation in the rings occur [11]. By reducing the post-prandial level of glucose in the bloodstream, flavonoids can therefore be used in diabetes treatment as substitutes for some drugs (e.g., acarbose) that cause side effects, such as abdominal distension, flatulence, meteorism, and diarrhoea [10].

In order to exert some of their physiological functions, polyphenols have to be absorbed in the gastrointestinal tract and reach the target tissue through the circulation system. So far, many studies have been conducted in order to elucidate the bioaccessibility of polyphenols, defined by Watson et al. [12] as the fraction of an ingested nutrient from the food matrix that is available for absorption. The bioaccessibility of polyphenols depends on several factors, which include their release from the food matrix, molecular size, hydrophilic/lipophilic balance as related to their glycosylation, and different pH-dependent transformations (degradation, hydrolysis, epimerization, and oxidation within the gastrointestinal tract) as well as solubility and interactions between polyphenols and food components [13]. In fact, polyphenols in liquid matrices are directly available for absorption while those in solid matrices have to first be extracted through mechanical, chemical, and enzymatic actions to make the absorption in the gastrointestinal tract easier [14].

Attention to the CBS has recently increased, and several studies [2,3] have focused on its uses as a source of bioactive compounds in food formulation. Numerous applications have been reviewed [3], mainly in order to take advantage of the antioxidant properties and high fibre content. In fact, CBS has been deemed suitable for use in the preparation of low-calorie and/or fibre-rich foods, such as chocolate biscuits, cakes, muffins, dietetic chocolate substitutes, and bread [3]. Furthermore, the antioxidant properties of the CBS were successfully tested to prevent lipid oxidation in cooked beef compared to synthetic BHT and β-tocopherol, and it has also been suggested for application in frying oils [3]. According to the literature, CBSs can also be used in the production of hot and cold beverages. Quijano-Aviles et al. [15] formulated a dairy drink enriched with CBSs, coffee silverskin, and orange peel aqueous extracts. Rojo-Poveda et al. [16] evaluated the functional properties of beverages obtained from CBSs with various particle sizes using six different extraction techniques (Mocha, Neapolitan, American, Espresso, Capsule, and French Press). In the latter work, encouraging results were obtained for the beverages from a nutritional point of view, with a high total phenolic content (up to 1803.83 mg GAE/L) and proven antioxidant and antidiabetic properties (up to 7.29 mmol of Trolox equivalents per litre of beverage and up to 52.00% of α-glucosidase inhibition, respectively). Nevertheless, there were still opportunities for further enhancements in consumer acceptance. In fact, the high content in polyphenols and methylxanthines and the acidity led to beverages with a bitter, astringent and unpleasant taste and flavour [16]. The addition of other ingredients beside CBS in the preparation would be beneficial to the taste, flavour and the overall liking of the beverages, especially if they are able to cover the acidity of the shell.

The main purpose of this study, as an extension of Rojo-Poveda et al.’s [16] study, was to assess the bioaccessibility of bioactive compounds and functional properties of two selected and sensorially enhanced beverages based on CBSs and enriched with different flavourings. In order to identify the most suitable beverages for potential commercialization, an evaluation of the sensory effects of the different flavouring combination together with a study of their polyphenolic content and functional properties was made prior to the bioaccessibility study.

## 2. Materials and Methods

### 2.1. Chemicals

Folin–Ciocalteu’s phenol reagent, 2-2′-diphenyl-1-picrylhydrazyl (DPPH), 6-hydroxy-2,5,7,8- tetramethylchroman-2-carboxylic acid (97%) (Trolox), methanol (≥99.9%), formic acid (≥98%), α-glucosidase from rat intestinal acetone powders, p-nitrophenyl-α-D-glucopyranoside (≥99%) (p-PNG), acarbose (≥95%), (+)-catechin hydrate (>98%), quercetin-3-*O*-glucoside (≥90%), theobromine (≥98.5%), caffeine (≥98.5%), α-amylase from *Bacillus* sp., pepsin from porcine gastric mucosa, pancreatin from porcine pancreas, and bile salts were supplied by Sigma-Aldrich (Milan, Italy). Sodium carbonate, potassium phosphate dibasic, potassium phosphate monobasic, potassium chloride, sodium bicarbonate, sodium chloride, magnesium chloride hexahydrate, ammonium carbonate, hydrochloric acid, and calcium chloride dihydrate were provided by Carlo Erba (Milan, Italy). All chemicals were of high purity. Gallic acid (≥98%), ethanol (≥99.9%), sodium hydroxide (1M), (–)-epicatechin (>90%), procyanidin B1 (PCB1) (≥98.5%), procyanidin B2 (PCB2) (≥98.5%), protocatechuic acid (>97%), and caffeic acid (≥95%) were provided by Fluka (Milan, Italy). Ultrapure water was prepared in a Milli-Q filter system (Millipore, Milan, Italy).

### 2.2. Samples

#### 2.2.1. Beverage Ingredients

The CBSs were obtained from Colombia roasted CBs (Criollo variety) that were kindly provided by Guido Gobino S.r.l. (Turin, Italy). They were ground with an Ultracentrifugal Mill ZM 200 (Retsch GmbH, Haan, Germany) and, according to Rojo-Poveda et al. [16], particle sizes of 500–1000 μm and 250–500 μm were chosen for the capsules and tea bags, respectively. The CBS composition, expressed as g/kg dry matter, was 178.0 protein, 34.0 fat, 106.0 carbohydrates, 545.0 dietary fibre, 68.0 water, and 69.0 ashes.

The CBs from Venezuela (Merida area and Criollo variety) were kindly provided by Venchi S.p.A. (Milan, Italy). The beans were ground and sieved (<1000 μm) with an Ultracentrifugal Mill ZM 200 (Retsch GmbH).

Turmeric, curry, vanillin, rooibos, coconut, mint, cinnamon, and liquorice, widely used as flavourings in tea and infusions for their intense aromas, were purchased from a local market in powdered form except for the coconut (grated), rooibos (dried leaves), and mint (dried leaves).

#### 2.2.2. Beverage Formulation

Thirteen formulations were prepared for each extraction method (Table 1). For each formulation, only one aromatic ingredient or a mix of two ingredients (at the same concentrations) were added to a mix of CBSs and CBs. The composition of mixes was defined according to other beverages on the market. The capsules were filled with 7 g of product while 3 g of product was used for the tea bags.

#### 2.2.3. Beverage Preparation

Among the extraction techniques tested by Rojo-Poveda et al. [16], the two most commonly used (capsules and tea bags) were selected to prepare the beverages.

An AEG capsule machine LM 3100 (Milan, Italy) was used for the capsule extraction. The capsules (SelfCap^®^; Mokitalia, Milan, Italy) were prepared according to Table 1, and 120 mL of beverage was obtained from each formulation in order to have a typical *lungo coffee*, which differs from the *espresso coffee* as it is less concentrated and requires a larger size of the cup (20–40 mL for the *espresso coffee* vs. 100–250 mL for the *lungo coffee*) [17]. The tea bags were produced with Teeli flip filters (Teeli^®^; Hamburg, Germany), filled according to Table 1, and infused in 100 mL of boiling water (100 °C) for 4 min. Natural mineral water (Valmora, Luserna San Giovanni, Italy) was used to prepare the beverages. Three independent beverages were prepared from each formulation.

For the analytical determinations, the obtained beverages were cooled in a dark room at 20 °C and centrifuged with a Heraeus Megafuge 11R centrifuge (Thermo Fisher Scientific, Waltham, MA, USA) at 7600× *g* for 5 min and then filtered through 0.45 μm cellulose acetate filter (Carlo Erba). The samples were kept at −20 °C until subsequent analysis.

### 2.3. Sensory Evaluation

Two sensory evaluations were performed. The first was a paired comparison test (ISO 5495:2005) used to compare the two concentrations of each tested aromatic ingredient. A two-sided difference test was used with α = 0.05, β = 0.5, and a pd = 40% and carried out by 24 untrained tasters (female = 70%; age range: 21–45 years). Participants received two cups with samples and were asked to rinse their mouths with still water before beginning the evaluation and in between samples. Beverages were served in a randomized and balanced order. The obtained results were evaluated according to ISO 5495:2005. The second test was a consumer acceptance test, which was conducted on 20 untrained tasters (female = 80%; age range: 21–65 years). Participants received individual cups with the samples and still water to rinse their mouths before beginning the evaluation and in between samples. Participants tasted the samples in a randomized and balanced order without any information about the beverage composition to avoid any potential bias on the liking scores. Participants rated their liking for “appearance”, “odour”, “taste”, “flavour”, and “overall liking” using a nine-point hedonic scale (1 = extremely dislike to 9 = extremely like [18]). “Purchase interest” was rated on a seven-point scale (1 = absolutely no to 7 = absolutely yes). Consumers took 3–10 min to complete the evaluation. Written informed consent was collected from all participants before the tests. The tests were performed in an air-conditioned room with white light at approximately 21 °C.

### 2.4. In Vitro Simulated Gastrointestinal Digestion (GID)

The most appreciated beverages, identified by the consumer acceptance test, underwent In Vitro GID. The digestion was carried out through a three-phase (oral, gastric, and intestinal) standardized protocol according to Minekus et al. [19]. Briefly, 5 mL of each selected beverage was mixed with simulated digestive fluids (simulated salivary fluid, simulated gastric fluid, and simulated intestinal fluid) consisting of the corresponding electrolyte stock solutions, enzymes, and water. Electrolyte stock solutions were previously heated in a SW-20 water bath (Julabo GmbH, Seelbach, Germany) at 37 °C. The digestion process was replicated three times for each beverage. A control, in which the sample was replaced by ultrapure water, was also prepared in triplicate in order to assess the contribution of digestion enzymes and simulated fluids in the subsequent analysis. Once the digestive phase was completed, the pH was brought down to 5.4 in order to stop the process. The samples were centrifuged at 12,500× *g* for 10 min at 4 °C, and the supernatants were passed through 0.45 μm cellulose acetate filters (Carlo Erba, Milan, Italy). The filtered samples were stored at 20 °C until subsequent analyses.

In Vitro bioaccessibility was calculated according to the following equation:

% Bioaccessibility = (C_POST_/C_PRE_) × 100 (1)

where C_POST_ and C_PRE_ correspond to the concentration after and before the digestion process, respectively.

### 2.5. Analytical Determinations

#### 2.5.1. Total Phenolic Content (TPC) Assay

The TPC of the beverages was determined according to the Folin–Ciocalteu colorimetric method adapted to a 96-well microplate [20] using a BioTek Synergy HT spectrophotometric multi-detection microplate reader (BioTek Instruments, Milan, Italy). All determinations were performed in triplicate. A calibration curve of standard gallic acid (100–500 μM) was built, and the results were expressed in mg of GAE per litre of beverage.

#### 2.5.2. Radical Scavenging Activity (RSA) Assay

RSA was determined by the DPPH method as reported by Von Gadow et al. [21] with slight modifications and adapted to a 96-well microplate [20]. The decrease in DPPH absorbance was measured at 515 nm in a BioTek Synergy HT microplate reader. All determinations were performed in triplicate. A Trolox standard curve (12.5–300 μM) was prepared for scavenging activity quantification, and the results were expressed in mmol of Trolox equivalents (TE) per litre of beverage.

#### 2.5.3. In Vitro α-Glucosidase Inhibition

The antidiabetic capacity of the beverages, evaluated as their α-glucosidase inhibition capacity, was determined by the α-glucosidase colorimetric assay detailed in Rojo-Poveda et al. [16], using a BioTek Synergy HT microplate reader. All measurements were run in triplicate. The antidiabetic capacity was expressed as percentage of α-glucosidase inhibition, and acarbose 0.5 mM (IC_50_) was used as the positive control.

#### 2.5.4. Bioactive Compound Analyses by Reverse-Phase Liquid Chromatography

Polyphenols and methylxanthines were determined by reverse-phase high pressure liquid chromatography coupled with photodiode array detector (RP-HPLC-PDA) as described by Rojo-Poveda et al. [16]. The samples were preliminarily filtered with PTFE membrane filters (LLG-Labware, Meckenheim, Germany) at 0.20 μm. The correlation coefficients of the external calibration curves obtained under the same chromatographic conditions and used for quantification were as follows: R^2^ = 0.9995 for theobromine, R^2^ = 0.9996 for caffeine, R^2^ = 0.9999 for catechin, R^2^ = 0.9998 for epicatechin, R^2^ = 0.9997 for protocatechuic acid, R^2^ = 0.9999 for caffeic acid, R^2^ = 0.9998 for PCB1, R^2^ = 0.9999 for PCB2, and R^2^ = 0.9996 for quercetin-3-*O*-glucoside. Catechin-3-*O*-glucoside and quercetin-3-*O*-rhamnoside were quantified as catechin and quercetin-3-*O*-glucoside equivalents, respectively.

### 2.6. Statistical Analysis

The results were statistically analysed with SPSS Statistics 25 software (IBM-SPSS Inc., Chicago, IL, USA). An analysis of variance (ANOVA) and Duncan’s post hoc test (95% confidence level) for TPC and RSA were used to compare the differences between mean values of the different formulations. The Kruskal-Wallis H-test (95% confidence level) with a multiple comparison test was applied for the consumer acceptance evaluation.

## 3. Results and Discussion

### 3.1. Comparison of the Beverages Based on Consumer Tests

A comparison test was carried out in order to select the best quantity of each aromatic ingredient for the capsules and tea bags. The obtained results (data not shown) highlighted that, for the formulation with turmeric and curry, the B1 mix with a lower quantity of aromatic ingredients was the most appreciated by tasters. The same result was obtained for F1 (mint) and G1 (cinnamon and liquorice mix) while for the other beverages, the higher quantity of ingredients was preferred (C2, D2, and E2). No differences (*p* > 0.05) were highlighted between the capsule and tea bag results, confirming that the selected quantities for each aromatic ingredient were the most appreciated by consumers.

Regarding the consumer test, Table 2 shows the results reported as the sum of ranks calculated for each beverage obtained by the capsule and tea bag methods.

Significant differences (*p* < 0.01) were found between the different formulations for all sensory parameters of both the capsule and tea bag methods with the exception of appearance, meaning that the different flavourings did not influence taster judgement on the look of the beverages, which resulted in equal rankings. This fact could be due to, among other possibilities, the small amount of the flavourings in the formulations compared to the other two main ingredients, namely CBSs and CBs, thus not having a strong impact on the visual aspect of the drink.

In capsule drinks, the coconut formulation (E2) obtained the highest score for all parameters with the exception of odour and appearance, in both of which it achieved the second highest score. On the other hand, formulations B1 (turmeric and curry) and F1 (mint) were the least appreciated by tasters with scores for odour, taste, flavour, overall liking, and purchase interest that were almost half of those characterizing formulation E2. Tea bag beverages showed a similar trend for capsule beverages, where the E2 formulation displayed the highest score for all parameters except appearance. Obtaining the highest scores, coconut turned out to be a valid ingredient to cover the acidity and bitterness of CBs and CBSs while also giving a tasteful flavour to the beverages. Coconut proved to be a valid ingredient to be combined with cocoa in other studies as well, gaining high levels of consensus among consumers [22,23]. For both the capsule and tea bag methods, formulation A, which contained only CBSs and CBs with no other flavouring, did not achieve the lowest score for all parameters with the exceptions of flavour and purchase predisposition in tea bag beverages. Thus, flavouring besides CBs did not always lead to an improvement of the beverage sensory characteristics as observed with formulations B1 and F1.

Comparing the two extraction techniques, capsule and tea bag beverages were not significantly different in most cases (*p* > 0.05) (Table S1 in Supplementary Materials). However, where significant differences (*p* < 0.05) were found, tea bags were characterized by a higher score. Hence, tea bags seemed to be more appreciated than capsules.

On the basis of the aforementioned reported results, the coconut formulation (E2) was used for further study of the bioaccessibility of bioactive compounds after In Vitro GID.

### 3.2. Total Phenolic Content (TPC) and Radical Scavenging Activity (RSA)

TPC and RSA were evaluated for the seven formulations selected using the paired comparison test. For all capsule and tea bag beverages, significant differences (*p* < 0.05) were found between the different formulations (Table 3).

For the capsule method, the formulations B1 (turmeric and curry), C2 (vanillin), and G1 (cinnamon and liquorice) displayed the highest content of TPC followed by formulations D2 (rooibos), F1 (mint), A (control), and E2 (coconut), which reported the lowest value. However, it should be highlighted that formulations A, B1, C2, F1, and G1 contained 97–100% CBSs and CBs while formulation E2 contained 80% of these two main ingredients, suggesting that coconut did not contribute to the TPC. Formulation D2 contained 90% CBSs and CBs but showed a high TPC due to the presence of rooibos. On the other hand, when tea bags were used, the highest TPC value was observed in formulation C2 followed by D2. For all other formulations (A, B1, E2, F1 and G2), no significant differences emerged (*p* > 0.05), thus presenting equivalent values.

Regarding antiradical activity, it is well known that one of the beneficial properties of polyphenols is their ability to react with free radicals as a result of the presence of hydroxyl groups [24]. Thus, the RSA is often correlated to TPC as confirmed by the present study with the exception of formulation C2 where vanillin was used. However, the high reactivity of vanillin with Folin–Ciocalteau’s reagent and its lack of response to the DPPH assay have been previously identified [25].

No significant differences (*p* > 0.05) were found between the two extraction methods for each formulation with the exceptions of formulations A, B1, and G1, which showed both higher TPC and RSA in capsule drinks. However, it should be noted that the two techniques used different quantities of ingredients and water (7 g in 120 mL for capsules and 3 g in 100 mL for tea bags). In fact, if the data are normalized on the grams of filling and volume of water used, tea bag beverages will show greater values than capsule drinks in almost all cases (Table S2 in Supplementary Materials) as demonstrated by Rojo-Poveda et al. [16]. This fact could be due to the lower solid/liquid ratio and longer time of contact between the ingredients and hot water (4 min vs. <30 s in tea bags and capsules, respectively) that occurred in the tea bag method. In fact, Ludwig et al. [26] and Gloess et al. [17] reported that a lower solid/liquid ratio and longer extraction time increased the extraction efficiency of bioactive compounds.

Compared with some of the drinks that can be found on the market, the formulated beverages have TPC values equivalent to or even higher than other infusions, such as mint (315 mg GAE/L), and fresh juice, including apple (339 mg GAE/L), pineapple (358 mg GAE/L), and white grape (519 mg GAE/L) [27]. Moreover, considering the TPC values normalized on the grams of filling obtained in this study (5.89–10.29 mg GAE/g and 10.05–19.54 mg GAE/g for capsules and tea bags, respectively, reported as the range from the lowest to the highest value among the formulations, Table S2 in Supplementary Materials), it can be observed that they are greater compared to that reported by Quijano-Aviles et al. [15] in an experimental dairy drink made of milk, CBSs, coffee husks, and orange peel (5.74 mg GAE/g).

### 3.3. Bioaccessibility of Bioactive Compounds and Functional Characteristics

The beverages with the highest values of overall liking—the E2 formulation in both capsules and tea bags—and therefore the greatest potential to be commercialized as final products underwent In Vitro GID in order to assess the bioaccessibility of their bioactive compounds, comparing the results obtained before digestion with those obtained after the digestion process. In particular, phenolic content through TPC and HPLC analyses (the latter for methylxanthines as well), RSA, and α-glucosidase inhibition capacity were evaluated.

#### 3.3.1. Determination of Polyphenol and Methylxanthine Composition of Undigested and Digested Beverages through RP-HPLC-PDA.

Table 4 shows the compounds detected in the beverages through liquid chromatography before and after GID (two methylxanthines and nine polyphenols). As far as polyphenols are concerned, phenolic acids (protocatechuic acid and caffeic acid), flavan-3-ols (catechin-3-*O*-glucoside, catechin and epicatechin), B-type procyanidins (PCB isomers), and flavonols (quercetin-3-*O*-glucoside and quercetin-3-*O*-rhamnoside) were identified.

In non-digested beverages (PRE), the most abundant compound was theobromine, a methylxanthine alkaloid that characterizes cocoa (72.34% and 74.89% of the total identified compounds for capsules and tea bags, respectively) followed by caffeine (17.18% and 14.24% of the total compounds identified for capsules and tea bags, respectively), another biologically active alkaloid. The total methylxanthine content was equal to 212.99 mg/L and 171.93 mg/L for capsules and tea bags, respectively. The ratio between these two alkaloids was approximately 5:1, which is in line with the data published by Rojo-Poveda et al. [16]. Concerning polyphenol composition, capsule and tea bag beverages showed the same profile with PCB2, PCB, and epicatechin as the most abundant compounds. In fact, for the total quantified polyphenols in capsule drinks, PCB2, PCB, and epicatechin were 25.47%, 19.75%, and 18.60% with respect to the total polyphenols quantified, respectively. Likewise, in tea bag drinks, PCB2, PCB, and epicatechin were 24.59%, 21.05%, and 18.45%, respectively.

After GID, the content of methylxanthines remained unchanged in both beverages, as they are stable under gastric and intestinal conditions and not degraded by pH and enzymes. Hence, the 5:1 ratio between theobromine and caffeine was maintained after GID. On the contrary, the concentration of polyphenols changed during GID with capsule and tea bag beverages showing the same behaviour. The total content of polyphenols quantified before GID was 24.95 and 20.99 mg/L for capsule and tea bag beverages, respectively. After GID, these values decreased, reaching a total of 12.45 and 11.56 mg/L with bioaccessibility of 50.00% and 55.46% for capsules and tea bags, respectively. These values represent the amount of soluble and accessible polyphenols not only to be absorbed but also to potentially exert their functions at the intestinal level, such as the ability to inhibit the α-glucosidase enzyme and the anti-inflammatory effects demonstrated by Rossin et al. [28]. The phenolic acids in both drinks degraded considerably with bioaccessibility that ranged from 37.43–38.21% for protocatechuic acid and 22.17–22.82% for caffeic acid. With regard to flavan-3-ols, in both capsule and tea bag beverages, catechin-3-*O*-glucoside degraded partially during GID (bioaccessibility of 47.41% and 55.75%, respectively) while epicatechin degraded almost completely (bioaccessibility of 10.55% and 12.50%, respectively). These results agree with the literature, which reports a poor availability of flavan-3-ols due to their instability in the gastrointestinal environment [13,29,30,31]. In fact, pH plays a key role in the stability of catechins and catechin glucosides, making them very unstable and rapidly subjected to degradation in neutral or alkaline solutions, whereas they are relatively stable in acidic solution [32]. Moreover, the high binding capacity of these compounds to digestive enzymes, which entail the polymerization and thus formation of insoluble aggregates, has been broadly reported [13,33]. The considerable loss of epicatechin during GID could therefore be explained by its high affinity for such enzymes and the mild alkaline milieu that typifies the intestinal phase. On the other hand, after GID, catechin content in capsule and tea bag beverages increased significantly (*p* < 0.01) with a bioaccessibility exceeding 100%. This boost could be derived from three different pathways. First, catechin-3-*O*-glucoside may have been hydrolysed in the intestinal phase, releasing aglycone. In fact, as reported by Raab et al. [32], catechin-3-*O*-glucoside shows a consistent degradation starting at pH 7 while it remains stable at low pH values. Hence, since glucoside shows a better resistance to degradation compared to aglycone [32], glycosylation could increase the bioaccessibility of catechin, which is delivered intact to the small intestine in an absorbable form. The second hypothesis is the epimerization of epicatechin into catechin under acidic conditions, as has been widely reported by many authors [34,35,36]. Lastly, a depolymerization of type B procyanidin with a consequent liberation of free catechin may have occurred, and this result could be confirmed by several studies on polyphenol bioaccessibility after GID [13,34,35,36]. Finally, regarding flavonols, both quercetin-3-*O*-glucoside and quercetin-3-*O*-rhamnoside degraded completely after In Vitro GID.

Comparing the beverages obtained with the two different extraction techniques, significant differences (*p* < 0.05) were found for protocatechuic acid after GID, catechin before GID, and caffeine and catechin-3-*O*-glucoside both before and after GID, which were found to be higher in capsules drink (Table 4). However, it should be highlighted that, once again, the solid/liquid ratio was different between capsules and tea bags (7 g of preparation in 120 mL of water in capsule beverages and 3 g in 100 mL in tea bag beverages). Thus, the results obtained from HPLC were normalized based on the grams of preparation contained in the capsules and tea bags (Table 5).

Considering the results, the tea bag method had a greater efficiency in extracting bioactive compounds (the reasons for which have already been discussed above), including 56.96% more methylxanthines, 63.64% more polyphenols, and 57.66% more total bioactive compounds. In fact, all compounds with the exceptions of caffeine and epicatechin were significantly higher in tea bags than capsules (*p* < 0.05).

Considering the quantity that may be consumed for one cup of each beverage (120 mL for capsules and 200 mL for tea bags), the dose intake of theobromine and caffeine would be 20.65 mg and 4.91 mg for capsules and 28.87 mg and 5.51 mg for tea bags, respectively, which also represents the potential amount available to be absorbed into the bloodstream. On the other hand, the dose intake of total polyphenols detected in this study would be 2.99 mg for capsules and 4.20 mg for tea bags with a potential post-GID availability of 1.49 mg and 2.31 mg, respectively. The U.S. Food and Drug Administration (FDA) has established 400 mg of caffeine per day in adults as a dose not generally related to dangerous and negative effects; however, this is contingent on individual sensitivity to the alkaloid and how fast it is metabolized [37]. Likewise, the European Food Safety Authority (EFSA) states that caffeine intake from all sources up to 400 mg per day (about 5.7 mg/kg body weight (bw) per day for a 70 kg adult) does not give rise to safety concerns for healthy adults with the exceptions of pregnant and lactating women, adolescents, and children, for whom the EFSA sets a limit of 200 mg per day (approximately 3 mg/kg bw per day) [38]. As for theobromine, firm conclusions have not been drawn yet. In fact, while in clinical studies of with a three- to four-week duration, dose levels of 150 mg theobromine/day (1.5–2.1 mg/kg bw) were well tolerated and adverse effects (such as nausea, vomiting, headache and diarrhoea) were only observed from doses higher than 500 mg theobromine/day, an actual level of no safety concern in humans has not yet been identified; however, the EFSA suggests that it is probably higher than 150 mg/day [39]. Based on these findings, the EFSA decided to derive its reference dose from the caffeine data since the results of pharmacokinetics studies of caffeine and its metabolites suggest that about 11% of caffeine oral intake is converted into theobromine and the two substances show a similar pharmacological profile [39]. Moreover, although the pharmacological effects of caffeine and theobromine can overlap, the latter shows a much lower potency than caffeine with respect to effects on the central nervous system, kidneys, or heart [39]. Therefore, the EFSA predicts a level of 0.6 mg/kg bw per day for healthy adults and 0.3 mg/kg bw per day for pregnant and lactating women, adolescents, and children to be of no safety concern [39] but also suggests that exceeding these doses would not necessarily result in a health risk. In view of this, considering that their values of caffeine and theobromine are lower than the recommended doses, both capsule and tea bag beverages could be considered safe for human health and are not expected to cause negative effects generally associated with overconsumption of these two methylxanthines, such as insomnia, anxiousness, tachycardia and nausea [37,39].

#### 3.3.2. TPC

**Figure 1:**
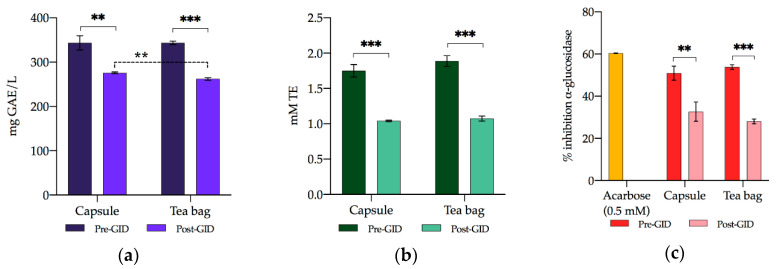
(**a**) TPC (mg GAE/L); (**b**) RSA (mM TE); (**c**) antidiabetic capacity (% α-glucosidase inhibition). For each panel results are reported before (Pre-Gastrointestinal Digestion (GID)) and after (Post-GID) GID for both capsule and tea bag coconut flavored beverages (E2), as well as results of ANOVA. Significance: ** = *p* < 0.01; *** = *p* < 0.001.

The TPC results are reported in Figure 1a. In digested beverages, the TPC was significantly lower compared to non-digested beverages. In fact, polyphenols are soluble in the matrix, and some of them are not very stable in the GID conditions due to degradation by enzymes, salts, and pH. However, the bioaccessibility of polyphenols in the capsule and tea bag beverages was still high at 80.32% and 76.28%, respectively. The bioaccessibility of polyphenols observed with this analytical method is not in line with that observed using HPLC, demonstrating once again that the methodology based on Folin–Ciocalteu’s reagent is not precise or reliable and tends to overestimate the result [40,41]. However, it should be noted that only the identified polyphenols were quantified by HPLC. Comparing the two different beverages (capsules vs. tea bags), no significant differences were found before GID. After GID, the TPC in capsule drinks was significantly (*p* < 0.01) higher than in tea bag drinks.

#### 3.3.3. RSA

The RSA significantly decreased (*p* < 0.001) as a result of the digestive process (Figure 1b), and the loss of activity was about 40.50% for capsules and 42.91% for tea bags. The greater loss of RSA relative to TPC can be ascribed to the nature of the phenolic compounds degraded after GID. In fact, it has been reported in the literature that RSA is highly controlled by the number and nature of the hydroxylation pattern on the aromatic ring of phenolic compounds: the more the hydroxyl groups on the aromatic ring, the more the antiradical activity [42]. In this way, each phenolic compound exhibits a higher or lower RSA depending on the redox properties of its hydroxyl groups and its potential for electron delocalization across the chemical structure [42]. The decrease in RSA could then be due to the almost complete degradation of caffeic acid, a hydroxycinnamic acid that is recognized to be one of the most effective scavengers among phenolic acids, followed by protocatechuic acid, a hydroxybenzoic acid with lower but still remarkable activity [42]. However, after GID, the free RSA remained considerable with a recovery of 59.50% for capsules and 57.09% for tea bags. No significant differences (*p* > 0.05) were found between the extraction methods before and after GID.

#### 3.3.4. α-Glucosidase Inhibition Capacity

In this study, the α-glucosidase inhibition capacity of the beverages was evaluated, and both capsule and tea bag drinks displayed a significant enzyme inhibition capacity (Figure 1c). It is interesting to observe that the concentration of polyphenols in the beverages obtained through the two systems (capsules and tea bags) is able to inhibit the enzyme to almost the same extent as 0.5 mM acarbose. As stated for TPC and RSA, during GID, a loss of activity occurred by 35.85% for capsules and 47.91% for tea bags. Since the α-glucosidase inhibition capacity is linked to the presence of polyphenols, the degradation of the latter during the digestive process led to the loss of enzyme inhibition capacity of the beverages. However, recovery of the inhibition activity was still appreciable (64.15% and 52.08% for capsules and tea bags, respectively). No significant differences emerged between the capsule and tea bag methods both before and after GID (*p* > 0.05). The flavoured beverages, both in the capsule and tea bag forms, before GID displayed almost twice as much enzyme inhibition activity as that found by Rojo-Poveda et al. [16]. This fact could be explained, among other things, with the most caffeic and catechin content in the flavoured beverages. In fact, it was reported that caffeic acid and catechin revealed to efficiently inhibit the α-glucosidase, especially catechin, which is able to induce 99.6% inhibition on the enzyme [43,44]. In recent years, daily consumption of cocoa or chocolate has been recommended in order to ensure an intake of flavanols, for which the role in protection against diabetes mellitus type 2 was proposed [45]. However, most of the cocoa products on the market contain little amounts of flavanols and are rich in fats and sugars, thus frustrating their potential protective effect and even worsening the disease [45]. Considering their composition, both the capsule and tea bag beverages could represent a natural and healthy approach to guarantee the intake of flavanols without the above-mentioned negative aspects.

## 4. Conclusions

The present study highlights the potential for CBSs to be used as a functional ingredient in the preparation of hot beverages obtained using two homemade extraction techniques: capsules and tea bags. The flavoured beverages were sensorially appreciated, especially the coconut-flavoured formulation, which turned out to be the most preferred by consumers. This formulation, in both the capsule and tea bag forms, showed a significant polyphenol content and was able to exert functional properties, such as antiradical and antidiabetic capacities. Moreover, the In Vitro bioaccessibility of polyphenols exceeded 50% in both capsule and tea bag beverages, highlighting their potential to be absorbed in the gastrointestinal tract or have beneficial effects at the intestinal level. Nonetheless, since In Vitro assessments often do not reflect what actually happens in the human body, further investigation would be needed to assess whether the bioaccessibility and the functional properties are also found *in vivo*. For these reasons, the present study displayed that CBSs can be turned into a health-promoting food ingredient, a valid alternative to its current uses (e.g., fuel, feed, and fertilizer).

## Figures and Tables

**Table 1:** Quantities (%) of CBS, cocoa beans (CB) and aromatic ingredients used for the production of beverages.

Formulation	CBS	CB	Turmeric	Curry	Vanillin	Rooibos	Coconut	Mint	Cinnamon	Licorice
A	97	3								
B1	94	3	1.5	1.5						
B2	91	3	3	3						
C1	96.6	3			0.4					
C2	96.1	3			0.9					
D1	92	3				5				
D2	87	3				10				
E1	87	3					10			
E2	77	3					20			
F1	95.5	3						1.5		
F2	94	3						3		
G1	95.4	3							0.8	0.8
G2	93.8	3							1.6	1.6

**Table 2:** Results of Kruskal–Wallis test (reported as sum of ranks) of consumer acceptance evaluation and results of the multiple comparison test.

Extraction Technique	Formulation	Appearance	Odor	Taste	Flavor	Overall Liking	Purchase Interest
Capsule	A	2672	2773 ^abc^	2777 ^abc^	2685 ^abc^	2717 ^ab^	3078 ^ab^
	B1	2753	2060 ^c^	1700 ^c^	2261 ^bc^	2021 ^b^	2050 ^b^
	C2	2803	3848 ^a^	2629 ^abc^	2407 ^abc^	2740 ^ab^	2627 ^ab^
	D2	3309	2577 ^bc^	3082 ^ab^	2994 ^abc^	2921 ^ab^	3090 ^ab^
	E2	3170	3533 ^ab^	3652 ^a^	3607 ^a^	3775 ^a^	3635 ^a^
	F1	2166	1813 ^c^	2343 ^bc^	2126 ^c^	2305 ^b^	2153 ^b^
	G1	2435	2311 ^c^	3123 ^ab^	3227 ^ab^	2829 ^ab^	2674 ^ab^
	*Sig.*	n.s.	***	***	**	***	***
Tea bag	A	2814	3134 ^ab^	2175 ^b^	2149 ^b^	2304 ^bcd^	2091 ^b^
	B1	2627	1666 ^c^	2012 ^b^	2154 ^b^	1985 ^d^	2197 ^b^
	C2	2027	3866 ^a^	3278 ^ab^	3163 ^ab^	3406 ^ab^	3689 ^a^
	D2	2831	1959 ^bc^	2562 ^b^	2411 ^b^	2267 ^bcd^	2257 ^b^
	E2	3394	3809 ^a^	3935 ^a^	3985 ^a^	3965 ^a^	3995 ^a^
	F1	2802	1707 ^c^	2260 ^b^	2256 ^b^	2098 ^cd^	2235 ^b^
	G1	2813	3166 ^ab^	3085 ^ab^	2798 ^b^	3282 ^abc^	2843 ^ab^
	*Sig.*	n.s.	***	***	***	***	***

Means followed by the same letter in the same column are not significantly different at *p* < 0.05 (multiple comparison test). Significance: ** = *p* < 0.01; *** = *p* < 0.001; n.s. = not significant.

**Table 3:** Values (mean ± standard deviation) of total phenolic content (TPC) and radical scavenging activity (RSA) for beverages obtained with capsule and tea bag extraction techniques and results of analysis of variance (ANOVA) with Duncan’s test performed between formulations for each extraction method (columns) and between extraction methods for each formulation (rows).

	Formulation	Capsule	Tea bag	*Sig.*
TPC (mg GAE/L)	A	384.98 ± 13.19 ^bc^	325.94 ± 26.43 ^c^	*
	B1	599.64 ± 62.14 ^a^	306.33 ± 12.86 ^c^	**
	C2	551.06 ± 50.37 ^a^	586.20 ± 57.27 ^a^	n.s.
	D2	455.18 ± 24.43 ^b^	476.93 ± 79.30 ^b^	n.s.
	E2	343.57 ± 15.46 ^c^	343.58 ± 4.77 ^c^	n.s.
	F1	431.55 ± 65.07 ^b^	339.39 ± 64.64 ^c^	n.s.
	G1	600.48 ± 25.22 ^a^	320.37 ± 28.43 ^c^	***
	*Sig.*	***	***	
RSA (mM TE/L)	A	1.97 ± 0.05 ^bc^	1.78 ± 0.12 ^b^	n.s.
	B1	2.92 ± 0.30 ^a^	1.70 ± 0.05 ^b^	**
	C2	2.01 ± 0.18 ^bc^	1.88 ± 0.04 ^b^	n.s.
	D2	2.31 ± 0.11 ^b^	2.53 ± 0.37 ^a^	n.s.
	E2	1.75 ± 0.08 ^c^	1.88 ± 0.09 ^b^	n.s.
	F1	2.21 ± 0.27 ^b^	1.96 ± 0.35 ^b^	n.s.
	G1	2.99 ± 0.25 ^a^	1.81 ± 0.09 ^b^	***
	*Sig.*	***	*	

GAE, gallic acid equivalents; TE, Trolox equivalents. Means followed by the same letter in the same column are not significantly different at *p* < 0.05. Significance: * = *p* < 0.05; ** = *p* < 0.01; *** = *p* < 0.001; n.s. = not significant.

**Table 4:** Values (mean ± standard deviation) of methylxanthines and identified polyphenols before (PRE) and after (POST) In Vitro gastrointestinal digestion for capsule and tea bag coconut flavored beverages (E2) and results of ANOVA. Significance is reported for each compound between PRE and POST In Vitro gastrointestinal digestion within the same extraction technique (column) and for each compound both PRE- and POST- In Vitro gastrointestinal digestion between the two extraction methods (row).

Extraction Method		Capsule	Tea Bag	*Sig.*
Formulation		E2	E2	
*Methylxanthines* *(mg/L of beverage)*				
Theobromine	PRE	172.09 ± 4.55	144.36 ± 18.83	n.s.
	POST	185.81 ± 27.59	150.70 ± 21.29	n.s.
	*Sig.*	n.s.	n.s.	
Caffeine	PRE	40.90 ± 2.02	27.57 ± 4.71	*
	POST	38.11 ± 5.62	25.97 ± 4.08	*
	*Sig.*	n.s.	n.s.	
*Polyphenols* *(mg/L of beverage)*				
*Phenolic acids*				
Protocatechuic acid	PRE	2.35 ± 0.14	1.93 ± 0.18	n.s.
	POST	0.90 ± 0.07	0.72 ± 0.08	*
	*Sig.*	***	***	
Caffeic acid	PRE	1.81 ± 0.14	1.51 ± 0.34	n.s.
	POST	0.40 ± 0.04	0.33 ± 0.02	n.s.
	*Sig.*	***	**	
*Flavan-3-ols*				
Catechin-3-*O*-glucoside	PRE	2.37 ± 0.08	1.94 ± 0.18	**
	POST	1.12 ± 0.13	1.08 ± 0.15	*
	*Sig.*	***	**	
Catechin	PRE	2.04 ± 0.11	1.65 ± 0.15	**
	POST	2.55 ± 0.25	2.71 ± 0.29	n.s.
	*Sig.*	**	***	
Epicatechin	PRE	4.64 ± 0.30	3.94 ± 1.17	n.s.
	POST	0.48 ± 0.22	0.47 ± 0.24	n.s.
	*Sig.*	***	**	
*Procyanidins type B*				
Procyanidin B isomer (PCB)	PRE	4.92 ± 0.56	4.41 ± 0.59	n.s.
	POST	4.21 ± 0.34	3.53 ± 0.49	n.s.
	*Sig.*	n.s.	n.s.	
Procyanidin B2 (PCB2)	PRE	6.36 ± 0.71	5.21 ± 1.22	n.s.
	POST	2.79 ± 0.38	2.72 ± 0.39	n.s.
	*Sig.*	**	*	
*Flavonols*				
Quercetin-3-*O*-glucoside	PRE	0.24 ± 0.03	0.23 ± 0.05	n.s.
	POST	n.d.	n.d.	n/a
	*Sig.*	n/a	n/a	
Quercetin-3-*O*-rhamnoside	PRE	0.21 ± 0.02	0.18 ± 0.03	n.s.
	POST	n.d.	n.d.	n/a
	*Sig.*	n/a	n/a	

n.d. = not detected. n/a = not applicable. Significance: * = *p* < 0.05; ** = *p* < 0.01; *** = *p* < 0.001; n.s. = not significant.

**Table 5:** Values (mean ± standard deviation) after normalization of methylxanthines and identified polyphenols before (PRE) and after (POST) In Vitro gastrointestinal digestion for capsule and tea bag coconut flavored beverages (E2) and results of ANOVA. Significance is reported for each compound between PRE- and POST-In Vitro gastrointestinal digestion within the same extraction technique (column) and for each compound both PRE- and POST-In Vitro gastrointestinal digestion between the two extraction methods (row).

Extraction Method		Capsule	Tea Bag	*Sig.*
Formulation		E2	E2	
*Methylxanthines* *(mg/g of filling)*				
Theobromine	PRE	2.95 ± 0.08	4.81 ± 0.63	**
	POST	3.19 ± 0.47	5.02 ± 0.71	*
	*Sig.*	n.s.	n.s.	
Caffeine	PRE	0.70 ± 0.03	0.92 ± 0.16	n.s.
	POST	0.65 ± 0.10	0.87 ± 0.14	n.s.
	*Sig.*	n.s.	n.s.	
*Polyphenols* *(μg/g of filling)*				
*Phenolic acids*				
Protocatechuic acid	PRE	40.35 ± 2.44	64.44 ± 7.53	**
	POST	15.38 ± 1.20	24.09 ± 2.53	**
	*Sig.*	***	**	
Caffeic acid	PRE	30.96 ± 2.42	50.25 ± 11.24	*
	POST	6.86 ± 0.64	11.08 ± 0.68	**
	*Sig.*	***	**	
*Flavan-3-ols*				
Catechin-3-*O*-glucoside	PRE	40.62 ± 1.30	64.74 ± 5.92	**
	POST	19.27 ± 2.20	36.07 ± 4.98	**
	*Sig.*	***	**	
Catechin	PRE	34.93 ± 1.86	55.06 ± 4.90	**
	POST	43.70 ± 4.37	90.26 ± 9.55	***
	*Sig.*	**	***	
Epicatechin	PRE	79.58 ± 5.12	131.18 ± 38.99	n.s.
	POST	8.25 ± 3.83	15.75 ± 8.04	*
	*Sig.*	***	**	
*Procyanidins* *B*				
Type B procyanidin	PRE	84.31 ± 9.55	146.94 ± 19.61	**
	POST	72.10 ± 5.86	117.58 ± 16.40	**
	*Sig.*	n.s.	n.s.	
Procyanidin B2	PRE	109.08 ± 12.18	173.58 ± 40.67	*
	POST	47.90 ± 6.49	90.58 ± 12.98	**
	*Sig.*	**	*	
*Flavonols*				
Quercetin-3-*O*-glucoside	PRE	4.19 ± 0.52	7.62 ± 1.66	*
	POST	n.d.	n.d.	n/a
	*Sig.*	n/a	n/a	
Quercetin-3-*O*-rhamnoside	PRE	3.60 ± 0.28	5.96 ± 0.86	*
	POST	n.d.	n.d.	n/a
	*Sig.*	n/a	n/a	

n.d. = not detected. n/a = not applicable. Significance: * = *p* < 0.05; ** = *p* < 0.01; *** = *p* < 0.001; n.s. = not significant.

## Supplementary Materials

The following are available online at https://www.mdpi.com/2304-8158/9/6/715/s1, Table S1: Results of comparison with Kruskal–Wallis test between capsule and tea bag for each formulation. Values are reported as sum of ranks, Table S2: Values (mean ± standard deviation) after normalization of total phenolic content (TPC) and radical scavenging activity (RSA) for beverages obtained with capsule and tea bag extraction techniques and results of ANOVA with Duncan’s test performed between formulations for each extraction method (columns) and between extraction methods for each formulation (rows).
