# Supplementary material for: In Vitro Bioaccessibility and Functional Properties of Phenolic Compounds from Enriched Beverages Based on Cocoa Bean Shell
Source: Foods. 2020 Jun 2;9(6):715. doi: 10.3390/foods9060715 (PMC7353536; doi:10.3390/foods9060715)
Supplement: Supplementary file 1 [file foods-09-00715-s001.pdf]

foods

MDPI

Article Supplementary Material

In vitro bioaccessibility and functional properties of phenolic compounds from enriched beverages based on cocoa bean shell

Carolina Cantele [1], Olga Rojo-Poveda [1,3], Marta Bertolino [1*], Daniela Ghirardello [1], Vladimiro Cardenia[1], Letricia Barbosa-Pereira [1,2], and Giuseppe Zeppa[1*]

[1] Department of Agricultural, Forest and Food Sciences (DISAFA), University of Turin, Grugliasco, Italy
[2] RD3 Department-Unit of Pharmacognosy, Bioanalysis and Drug Discovery, Faculty of Pharmacy, Université libre de Bruxelles, Brussels, Belgium
[3] Department of Analytical Chemistry, Nutrition and Food Science, Faculty of Pharmacy, University of Santiago de Compostela, Santiago de Compostela, Spain

* Correspondence: marta.bertolino@unito.it; giuseppe.zeppa@unito.it; Tel.: +39-011-670-8686 (M.B.) +39-011-670-8705 (G.Z.)

Received: date; Accepted: date; Published: date

Abstract: The cocoa bean shell (CBS), a cocoa by-product, contains a significant amount of bioactive compounds with functional properties, such as polyphenols and methylxanthines, and is used as an ingredient in beverages and foods. In this work, the bioaccessibility of polyphenols and methylxanthines after *in vitro* digestion was evaluated in new flavoured beverages for at-home consumption (capsules and tea bags). In addition, the polyphenolic composition, functional properties (antiradical and α-glucosidase inhibition capacities) and consumer acceptability of these beverages were evaluated. In both capsule and tea bag beverages, the bioaccessibility of methylxanthines was 100% while that of total polyphenols exceeded 50%. The main polyphenols determined using reverse-phase liquid chromatography were type B procyanidins and epicatechin. The antiradical activity in capsule and tea bag beverages was 1.75 and 1.88 mM of Trolox equivalents, respectively, of which 59.50% and 57.09% were recovered after simulated digestion. The percentage of α-glucosidase inhibition before *in vitro* digestion (51.64% and 53.82% for capsules and tea bags, respectively) was comparable to that of acarbose at 0.5 mM. All the beverages obtained a high consumer acceptability. Therefore, these results highlight that CBSs can be used as a valid source of bioactive compounds in the preparation of beverages with homemade techniques.

Keywords: cocoa bean shell; by-products; polyphenols; bioaccessibility; capsule; tea bag; antiradical activity; α-glucosidase inhibition

Table S1. Results of comparison with Kruskall-Wallis test between capsule and tea bag for each formulation. Values are reported as sum of ranks.

Formulation		Appearance	Odor	Taste	Flavor	Overall liking	Purchase interest
A	Capsule	2672	2773	2777	2685	2717	3078
	Tea bag	2814	3134	2175	2149	2304	2091
	Sig.	*	n.s.	n.s.	n.s.	n.s.	n.s.
B1	Capsule	2753	2060	1700	2261	2021	2050
	Tea bag	2627	1666	2012	2154	1985	2197
	Sig.	n.s.	n.s.	*	n.s.	n.s.	n.s.
C2	Capsule	2803	3848	2629	2407	2740	2627
	Tea bag	2027	3866	3278	3163	3406	3689
	Sig.	n.s.	n.s.	**	**	**	***
D2	Capsule	3309	2577	3082	2994	2921	3090
	Tea bag	2831	1959	2562	2411	2267	2257
	Sig.	n.s.	*	n.s.	n.s.	n.s.	n.s.
E2	Capsule	3170	3533	3652	3607	3775	3635
	Tea bag	3394	3809	3935	3985	3965	3995
	Sig.	n.s.	n.s.	*	*	n.s.	n.s.
F1	Capsule	2166	1813	2343	2126	2305	2153
	Tea bag	2802	1707	2260	2256	2098	2235
	Sig.	**	n.s.	n.s.	*	n.s.	n.s.
G1	Capsule	2435	2311	3123	3227	2829	2674
	Tea bag	2813	3166	3085	2798	3282	2843
	Sig.	n.s.	n.s.	n.s.	n.s.	**	*

Significance: ** = $p < 0.01$; *** = $p < 0.001$; n.s. = not significant.

Table S2. Values (mean ± standard deviation) after normalization of total phenolic content (TPC) and radical scavenging activity (RSA) for beverages obtained with capsule and tea bag extraction techniques and results of ANOVA with Duncan's test performed between formulations for each extraction method (columns) and between extraction methods for each formulation (rows).

	**Formulation**	**Capsule**	**Tea bag**	***Sig.***
TPC (mg GAE /g of filling)	A	6.60±0.23 [bc]	10.86±0.88 [c]	**
	B1	10.28±1.07 [a]	10.21±0.43 [c]	n.s.
	C2	9.45±0.86 [a]	19.54±1.91 [a]	**
	D2	7.80±0.42 [b]	15.90±2.64 [b]	*
	E2	5.89±0.26 [c]	10.05±1.42 [c]	**
	F1	7.40±1.12 [b]	11.31±2.15 [c]	*
	G1	10.29±0.43 [a]	10.68±0.95 [c]	n.s.
	Sig.	***	***	
RSA (µmol TE /g of filling)	A	33.72±0.86 [bc]	59.25±3.84 [b]	**
	B1	50.12±5.16 [a]	56.78±1.53 [b]	n.s.
	C2	34.50±3.05 [bc]	62.67±1.29 [b]	***
	D2	39.67±1.94 [b]	84.19±12.27 [a]	**
	E2	30.02±1.33 [c]	62.38±3.55 [b]	***
	F1	37.90±4.68 [b]	65.40±11.81 [b]	*
	G1	51.18±4.27 [a]	60.28±3.14 [b]	*
	Sig.	***	**	

GAE, gallic acid equivalents; TE, Trolox equivalents.
Means followed by the same letter in the same column are not significantly different at $p<0.05$.
Significance: * = $p < 0.05$; ** = $p < 0.01$; *** = $p < 0.001$; n.s. = not significant.

© 2020 by the authors. Submitted for possible open access publication under the terms and conditions of the Creative Commons Attribution (CC BY) license (http://creativecommons.org/licenses/by/4.0/).
